# The Enhanced Expression of Cruzipain-Like Molecules in the Phytoflagellate *Phytomonas serpens* Recovered From the Invertebrate and Plant Hosts

**DOI:** 10.3389/fcimb.2021.819133

**Published:** 2022-01-13

**Authors:** Simone S. C. Oliveira, Camila G. R. Elias, Felipe A. Dias, Angela H. Lopes, Claudia M. d’Avila-Levy, André L. S. Santos, Marta H. Branquinha

**Affiliations:** ^1^ Laboratório de Estudos Avançados de Microrganismos Emergentes e Resistentes (LEAMER), Instituto de Microbiologia Paulo de Góes (IMPG), Departamento de Microbiologia Geral, Universidade Federal do Rio de Janeiro (UFRJ), Rio de Janeiro, Brazil; ^2^ Laboratório de Bioquímica de Microrganismos, Instituto de Microbiologia Paulo de Góes (IMPG), Departamento de Microbiologia Geral, Universidade Federal do Rio de Janeiro (UFRJ), Rio de Janeiro, Brazil; ^3^ Laboratório de Estudos Integrados em Protozoologia, Instituto Oswaldo Cruz, Fundação Oswaldo Cruz (FIOCRUZ), Rio de Janeiro, Brazil; ^4^ Programa de Pós-Graduação em Bioquímica (PPGBq), Instituto de Química, Universidade Federal do Rio de Janeiro (UFRJ), Rio de Janeiro, Brazil

**Keywords:** *Phytomonas serpens*, tomato, *Oncopeltus fasciatus*, invertebrate vector, interaction, cruzipain

## Abstract

*Phytomonas serpens* is a protozoan parasite that alternates its life cycle between two hosts: an invertebrate vector and the tomato fruit. This phytoflagellate is able to synthesize proteins displaying similarity to the cysteine peptidase named cruzipain, an important virulence factor from *Trypanosoma cruzi*, the etiologic agent of Chagas disease. Herein, the growth of *P. serpens* in complex medium (BHI) supplemented with natural tomato extract (NTE) resulted in the increased expression of cysteine peptidases, as verified by the hydrolysis of the fluorogenic substrate Z-Phe-Arg-AMC and by gelatin-SDS-PAGE. Phytoflagellates showed no changes in morphology, morphometry and viability, but the proliferation was slightly reduced when cultivated in the presence of NTE. The enhanced proteolytic activity was accompanied by a significant increase in the expression of cruzipain-like molecules, as verified by flow cytometry using anti-cruzipain antibodies. In parallel, parasites incubated under chemically defined conditions (PBS supplemented with glucose) and added of different concentration of NTE revealed an augmentation in the production of cruzipain-like molecules in a typically dose-dependent way. Similarly, *P. serpens* recovered from the infection of mature tomatoes showed an increase in the expression of molecules homologous to cruzipain; however, cells showed a smaller size compared to parasites grown in BHI medium. Furthermore, phytoflagellates incubated with dissected salivary glands from *Oncopeltus fasciatus* or recovered from the hemolymph of infected insects also showed a strong enhance in the expression of cruzipain-like molecules that is more relevant in the hemolymph. Collectively, our results showed that cysteine peptidases displaying similarities to cruzipain are more expressed during the life cycle of the phytoflagellate *P. serpens* both in the invertebrate and plant hosts.

## Introduction

The genus *Phytomonas* (Kinetoplastea: Trypanosomatidae) was first mentioned in the early 1900s when proposed a classification for flagellate trypanosomatids found in plants (Camargo, 1999; Jaskowska et al., 2015). Infection by *Phytomonas* spp. can be associated to pathological syndromes in plants of great economic importance such as coffee, cassava, coconut and oil palm (Dollet, 1984; Camargo, 1999). On the other hand, in many cases flagellates parasitize plants without apparent pathogenicity (Camargo, 1999; Jaskowska et al., 2015). *Phytomonas serpens* is a flagellate isolated for the first time in the sap of tomato fruits (*Solanum lycopersicum*) (Gibbs, 1957; Jankevicius et al., 1989). There is no precise available information about the pathogenicity of trypanosomatids in edible fruits such as tomatoes, oranges and grapes, although flagellates usually remain limited around the point of inoculation. When it comes to *P. serpens*, the presence of these trypanosomatids provoke only yellowish spots on the fruit surface that are not ascertained to be real injuries to tomato; however, a loss in nutritional quality and, above all, a loss in economic value added to the product are undoubtedly documented (Camargo, 1999).

*Phytomonas serpens* is transmitted to tomatoes through the bite of hemipteran phytophagous insects, such as *Phthia picta* and *Nezara viridula* (Jankevicius et al., 1989; Camargo, 1999). In insects, ingested parasites initially colonize intestinal sites, where the first multiplication of *P. serpens* cells occurs. The parasites then migrate through hemolymph to salivary glands, where they resume proliferation (Mcghee and Hanson, 1964). Transmission occurs when insects with infected salivary glands feed on the fruit (Jankevicius et al., 1989; Camargo and Wallace, 1994).

Among trypanosomatids, *P. serpens* represents an excellent biological study model due to the expression of molecules similar to those described in human pathogenic species (Jaskowska et al., 2015). The parasite expresses a 63-kDa cell surface polypeptide that is similar to the main metallopeptidase present in *Leishmania* spp, called gp63 or leishmanolysin; however, these molecules do not show any proteolytic activity in the phytoflagellate (Breganó et al., 2003; Santos et al., 2007; d'Avila-Levy et al., 2014). Breganó et al. (2003) demonstrated that *P. serpens* displays antigens similar to those of *Trypanosoma cruzi*, which were strongly recognized by the serum of patients with Chagas disease besides being capable of providing protective immunity in susceptible BALB/c mice. Subsequently, our group identified in *P. serpens* the presence of two 38- and 40-kDa cysteine-type peptidases that have similar antigenic properties to the main cysteine peptidase of *T. cruzi*, called cruzipain (Santos et al., 2006; Santos et al., 2007; Elias et al., 2012). This finding is directly related to the strong reactivity of *P. serpens* antigens with sera from patients with Chagas disease (Santos et al., 2007).

The important roles of cysteine peptidases in *P. serpens* are strengthened by the modulation of their expression when the parasite is cultured in different culture media. Elias and collaborators (Elias et al., 2008) reported a strong reduction in the 38- and 40-kDa peptidase activities when parasites were grown in either liver infusion trypticase or yeast extract media, when compared to parasites grown in Warren medium. Additionally, cruzipain-like molecules were differentially modulated according to the proteins present in the culture medium (Elias et al., 2008). Cultivation of *P. serpens* in phosphate-buffered saline (PBS)-glucose supplemented with human and bovine albumins led to a reduction in the expression of these proteins by about 50% and 25%, respectively, while mucin and fetal bovine serum did not alter their production. In contrast, immunoglobulin G (IgG) and hemoglobin drastically enhanced its surface expression by about 7- and 11-fold, respectively, when compared with parasites incubated in PBS-glucose (Elias et al., 2012). Incubation of *P. serpens* in PBS-glucose supplemented with hemoglobin at different concentrations also induced a dose-dependent increase in the activity of both cysteine peptidases; in addition, a significant increase in the secretion of cruzipain-like molecules was also observed after incubation of the parasites with 1% hemoglobin, in comparison with parasites incubated in PBS-glucose (Elias et al., 2012).

With these premises in mind, the present study aims to evaluate and compare the modulation of the expression of cruzipain-like cysteine peptidases in the presence of natural tomato extract (NTE), as tomato is the original plant host of *P. serpens*, as well as during the interaction process with the plant host and with the phytophagous hemipteran *Oncopeltus fasciatus*, usually employed as an invertebrate host model for studies of this parasite (Santos et al., 2006; Dias et al., 2012).

## Materials and Methods

### Parasite and Growth Conditions

*Phytomonas serpens* (isolate 9T; CT-IOC-189), isolated from tomatoes, was provided by Coleção de Tripanossomatídeos, Instituto Oswaldo Cruz – Fundação Oswaldo Cruz, Rio de Janeiro, Brazil. Promastigote forms (1 × 10^6^ parasites/ml) were grown in 3.7% (w/v) brain–heart infusion (BHI) medium supplemented with 10% (v/v) heat-inactivated fetal bovine serum (FBS) at 26°C for 48 h. Cellular growth was estimated by counting the parasites in a Neubauer chamber. The cellular viability was monitored through parasite motility (Elias et al., 2008).

### Preparation of NTE Medium

Fresh and mature tomatoes of the species *Solanum lycopersicum* were washed in a solution of distilled water with 0.01% sodium hypochlorite. Then, tomatoes were macerated, weighted and diluted in PBS (150 mM NaCl, 20 mM phosphate buffer, pH 7.2) to a final concentration of 3 g/ml. After dilution, the extract was centrifuged at 3,500×g for 30 min. The supernatant, called NTE, was removed and subjected to sterilization through filtration on a Millipore 0.22-µm membrane.

### Cell Morphology and Ultrastructural Analysis

In order to detect morphological alterations, *P. serpens* promastigotes were grown in BHI medium supplemented or not with 25% (BHI-NTE 25%) or 50% NTE (BHI-NTE 50%) for 48 h at 26°C. Then, the parasites were washed three times in PBS, fixed with methanol for 5 min, stained with Giemsa and then observed under a Zeiss microscope (Axioplan, Oberkochen, Germany). In parallel, each experimental population was mapped by flow cytometry (FACSCalibur, BD Bioscience, USA) using a two-parameter histogram of forward scatter (FSC) versus side scatter (SSC) to measure two morphometric parameters: cell size and granularity, respectively. At the same time, aliquots were collected from each culture medium before inoculation (0 h) and after 48 h of growth to measure pH values over time. Ultrastructural analysis was performed by scanning electron microscopy (SEM), in which promastigotes were cultivated under the same conditions and then fixed for 40 min at 25°C with 2.5% glutaraldehyde in 0.1 M cacodylate buffer, pH 7.2. After fixation, cells were washed in cacodylate buffer and postfixed with a solution of 1% OsO_4_, 0.8% potassium ferrocyanide and 5 mM CaCl_2_ in the same buffer for 20 min at 25°C. Cells were dehydrated in graded series of acetone (30–100%) and then dried by the critical point method, mounted on stubs, coated with gold (20–30 nm) and observed in a Jeol JSM 6490LV scanning electron microscope (Massachusets, USA).

### Parasite Extracts

*Phytomonas serpens* promastigotes grown in BHI medium supplemented or not with 25% or 50% NTE for 48 h were harvested by centrifugation for 5 min at 500×g at 4°C and washed three times with cold PBS. Then, parasites were lysed by the addition of 1% Triton X-100 (Sigma Aldrich, St Louis, USA) and homogenized using a vortex by alternating 30-seg shaking and 1-min cooling intervals. Then, the cellular extract was centrifuged at 1,500×g for 15 min at 4°C. Protein concentration was determined by the method described by Lowry et al. (1951), using bovine serum albumin (BSA) as standard.

### Cysteine Peptidase Activity Assays

Cysteine peptidase activity was determined using *P. serpens* promastigotes grown in the absence (control) or in the presence of 25% or 50% of NTE. Cellular parasite extracts were obtained as described in the previous item. The fluorogenic peptide substrate *N*-benzyloxycarbonyl-L-phenylalanyl-L-arginine-(7-amino-4-methylcoumarin) (Z-Phe-Arg-AMC) (Sigma Aldrich, St Louis, USA) was used as a specific cysteine peptidase substrate. The 5-mM stock solution of the fluorogenic substrate was prepared in DMSO. The reaction was started by the addition of the substrate (20 μM) to the parasite extract (10 μg protein) by diluting in 0.1M sodium phosphate buffer, pH 5.0, containing 2 mM dithiothreitol (DTT). The reaction mixture was incubated at 37°C for 15 min and the clearance of the fluorogenic substrate was monitored continuously by a spectrofluorometer (SpectraMax Gemini XPS, Molecular Devices, CA, USA) using emission and excitation wavelengths of 460 and 380 nm, respectively. Concomitantly, cysteine peptidase activities were assayed in gelatin-containing sodium dodecyl sulfate-polyacrylamide gel (SDS-PAGE) (Heussen and Dowdle, 1980). Samples containing 50 µg protein from each system were resuspended in SDS-PAGE sample buffer (125 mM Tris, pH 6.8, 4% SDS, 20% glycerol and 0.002% bromophenol blue). Peptidases were assayed and characterized by 10% SDS-PAGE with 0.1% co-polymerized gelatin as substrate. After electrophoresis at a constant voltage of 120 V at 4°C, SDS was removed by incubation with 2.5% Triton X-100 for 1 h at room temperature under constant agitation. Then, gels were incubated at 37°C in sodium phosphate buffer, pH 5.0, supplemented with 2 mM DTT for 48 h in the absence or in the presence of the following cysteine peptidase inhibitors: cystatin (1 μM), leupeptin (1 μM), antipain (1 μM), iodoacetamide (1 μM) and E-64 (1 µM). After incubation, gels were washed twice with distilled water, and stained for 24 h with 0.2% Coomassie brilliant blue R-250 in methanol-acetic acid-water (50:10:40) and destained overnight in a solution containing methanol-acetic acid-water (5:10:85). The molecular masses of the peptidases were estimated by comparison with the mobility of low molecular mass standards (Thermo Fisher Scientific, Massachusetts, USA). The densitometric analysis was performed using the ImageJ program.

## Detection of Cruzipain-Like Molecules

### Modulation of Expression by NTE

Promastigotes of *P. serpens* were cultivated in BHI medium supplemented or not with 25% or 50% NTE for 48 h. Alternatively, parasites (8 × 10^8^ cells) were incubated in PBS-glucose 2% supplemented or not with 0.1, 1 or 10% NTE for 3 h at room temperature. After this period, parasites were centrifuged and cells were fixed for 15 min in 2% paraformaldehyde in PBS (pH 7.2) at room temperature, and then washed with the same buffer. Morphological integrity was verified by optical microscopic observation. Cells were incubated for 1 h at room temperature with a 1:250 dilution of rabbit anti-cruzipain polyclonal antibody raised against *T. cruzi* (kindly provided by Dr Juan-Jose Cazzulo, Instituto de Investigaciones Biotecnologicas, Universidad Nacional de General San Martin, Buenos Aires, Argentina). Then, cells were incubated for an additional hour with a 1:100 dilution of fluorescein isothiocyanate (FITC)-labeled goat anti-rabbit immunoglobulin G (IgG) (Sigma Aldrich, St Louis, USA). Finally, cells were washed three times in PBS and analyzed in a flow cytometry (FACSCalibur, BD Bioscience, USA) equipped with a 15-mW argon laser emitting at 488 nm. Non-treated cells and those treated with the secondary antibody alone were run in parallel as controls (d'Avila-Levy et al., 2006). The results were expressed as the percentage of fluorescent cells (%FC) and mean of fluorescence intensity (MFI).

### Modulation of Expression by Tomato Fruit (*Solanum lycopersicum*)

Parasites cultivated in BHI medium were collected by centrifugation, washed in PBS and counted in a Neubauer chamber. Concomitantly, tomatoes were initially treated for 15 min in a solution of chlorine and water (20 ppm) and left for 30 min under ultraviolet light. Then, parasites (5 × 10^4^ cells resuspended in 5 μl PBS) were inoculated in 3 sites previously chosen in tomatoes. Fruits were incubated for seven days at 26°C, during which no signs of decomposition were visible. Finally, tomatoes were cut, seeds separated and parasites removed by endocarp filtration. Parasites were then collected and prepared for flow cytometry using anti-cruzipain antibody, as described previously.

### Modulation of Expression by the Salivary Glands of the Phytophagous Insect *O. fasciatus*

A milkweed bug (*O. fasciatus*) culture was purchased from Carolina Biological Supply Company, Burlington, North Carolina, USA and originated the colony maintained in our department. Adult *O. fasciatus* (Hemiptera: Lygaeidae) were kept in plastic pitchers under a 12 h light/dark cycle at 28°C with 70-80% relative humidity and fed with peeled and toasted sunflower seeds and distilled water (Romeiro et al., 2000). Salivary glands of *O. fasciatus* were carefully dissected, washed three times in ice-cold PBS and resuspended in PBS-glucose 2%. In parallel, parasites cultivated in BHI for 48 h were collected by centrifugation, washed in PBS and counted in a Neubauer chamber. Then, parasites (8 × 10^8^ cells) were incubated with dissected salivary glands for 3 h (Santos et al., 2006). After incubation, parasites were collected by centrifugation and treated with anti-cruzipain antibody and analyzed by flow cytometry, as described previously. Control systems were used with parasites incubated only in PBS-glucose 2%.

### Modulation of Expression by the Hemolymph of *O. fasciatus*

Parasites were grown for 48 h in BHI, washed three times in PBS and counted in a Neubauer chamber. Then, parasites were resuspended in PBS (4 × 10^5^ cells) and injected, with a Hamilton syringe, on the side of the junction between the second and third thoracic segments of *O. fasciatus*. After 72 h of infection, hemolymph was collected by clipping off the first two pairs of insect legs (Alves e Silva et al., 2013). Then, hemolymph was centrifuged at 5,000×g for 10 min to separate parasites, which were washed in PBS and processed for analysis by flow cytometry using the anti-cruzipain antibody. After incubation, the parasites were collected by centrifugation and treated with anti-cruzipain antibody and analyzed by flow cytometry, as described previously.

### Statistics

All experiments were performed in triplicate, in three independent experimental sets. Results were analyzed statistically by Student’s t-test (in the comparisons between two groups) and by Analysis of Variance One-Way ANOVA followed by a Tukey-Kramer post-test (in comparisons between three or more groups). In all analyses, *p* values of 0.05 or less were considered statistically significant. All analyzes were performed using the program GraphPad Prism version 6.0 (GraphPad Software, San Diego, CA, USA).

## Results

### Effects of NTE on the Growth Rate and Morphology of *P. serpens*

**Figure 1:**
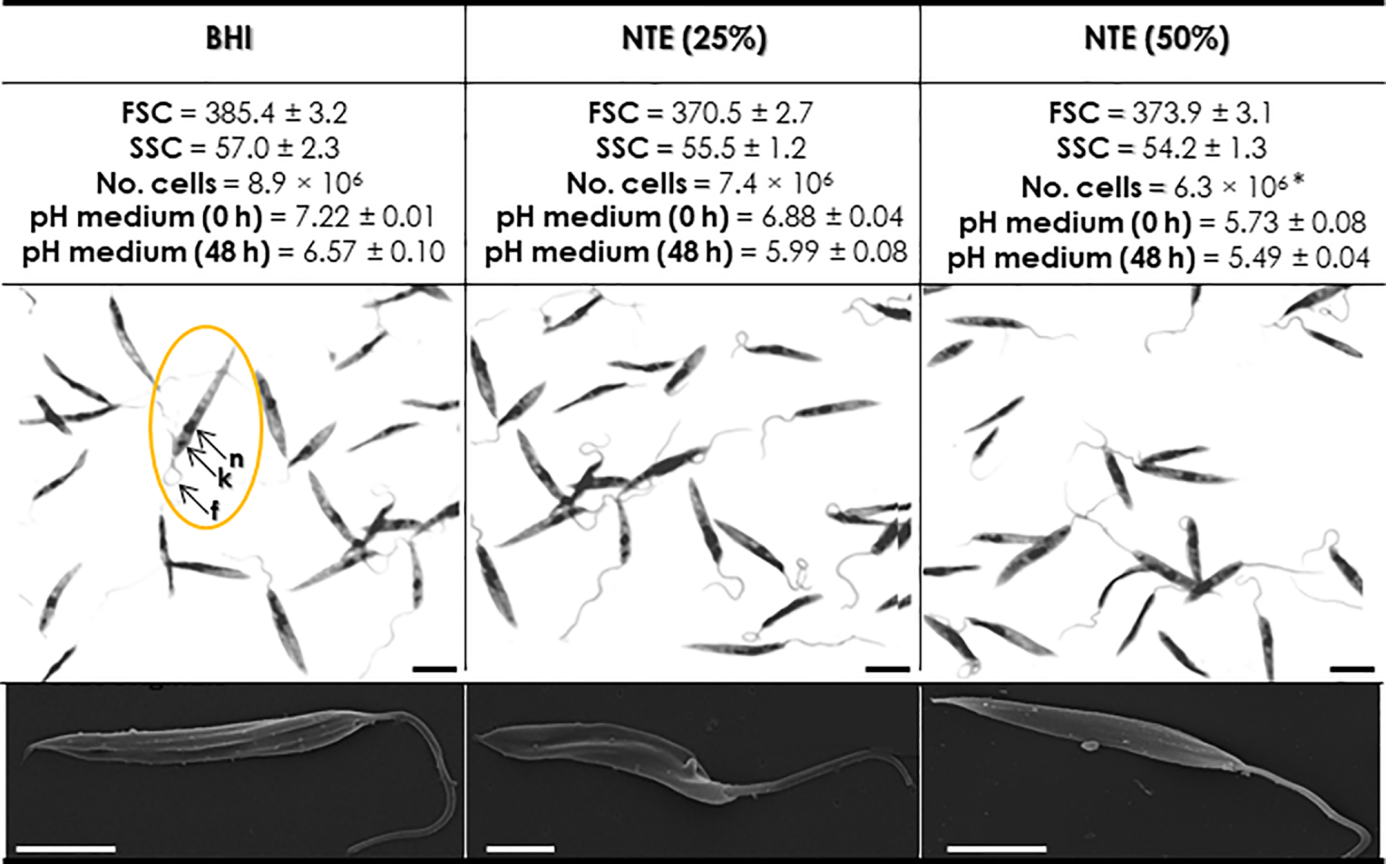
Effects of natural tomato extract (NTE) on *Phytomonas serpens* morphology and growth after cultivation in BHI medium for 48 h in the absence (BHI) or in the presence of different concentrations of NTE (25 and 50%). pH values were also measured in each culture medium before inoculation (0 h) and after 48 h of growth. Cells were analyzed by flow cytometry in order to measure two morphometric parameters, forward scatter (FSC) and side scatter (SSC). The values expressed represent the mean of fluorescence intensity of three independent experiments. The symbol (*) represents the significant difference (*P* < 0.05; Student’s *t*-test) between treated and control groups. The influence of NTE was also evaluated during cell proliferation (initial inoculum of 1 x 10^6^ parasites/mL) by counting cells in a Neubauer chamber after 48 h. In parallel, Giemsa-stained smears were analyzed under an optical microscope. Promastigote forms present a kinetoplast (k), the central nucleus (n), an elongated cell body and a flagellum (f) attached to the parasite cell body. Bars: 10 µM. In scanning electron microscopy analysis of promastigotes, note that no morphological changes were identified after growth of parasites in these different media. Bars: 5 µm.

The first step of this study was to evaluate the effect of NTE on *P. serpens* morphology and growth. Analysis by flow cytometry showed that parasites grown in BHI-NTE 25% and BHI-NTE 50% for 48 h presented similar cell size and granularity when compared to parasites grown in the standard BHI medium, as revealed by both FSC and SSC flow cytometry parameters (**Figure 1**). Corroborating this result, both light and scanning electron microscopies revealed no morphological differences between these populations, in which *P. serpens* had the typical promastigote form presenting an elongated cell body, an anterior kinetoplast in relation to the nucleus and a single long flagellum (**Figure 1**). The presence of NTE in the culture medium was also able to reduce the proliferation rate of *P. serpens* in a dose-dependent manner: the cultivation in BHI-NTE 25% promoted a reduction of approximately 17%, while the cultivation in BHI-NTE 50% of around 30% (**Figure 1**). Before inoculation, pH values in the different culture media showed that the higher the concentration of NTE, the lower the pH value; after 48 h of culture, there was an acidification of BHI medium and, more prominently, in BHI-NTE 25% medium. The pH of BHI-NTE 50% medium remained practically constant after 48 h of growth (**Figure 1**).

### Effects of NTE on the Proteolytic Activities of *P. serpens*

**Figure 2:**
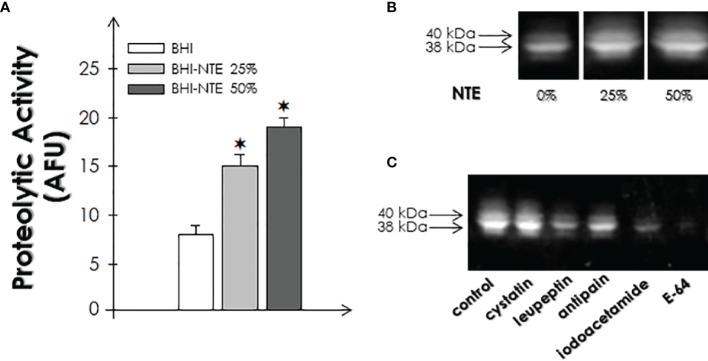
Effects of natural tomato extract (NTE) on cysteine peptidase activity in promastigotes of *P. serpens* cultivated in BHI medium for 48 h in the absence or in the presence of different concentrations of NTE (25 and 50%). **(A)** Cysteine ​​peptidase activity in parasite lysates was assessed by measuring the hydrolysis of Z–Phe–Arg–AMC. Results are expressed as arbitrary fluorescence units (AFU). Data shown are the mean ± standard deviation (SD) of three independent experiments performed in triplicate. Symbols denote statistical differences (P < 0.05; Student’s *t*-test) between cells grown in BHI medium and cells grown in BHI-NTE medium (25% or 50%). **(B)** The peptidase profiles in cell extracts were analyzed by means of gelatin-SDS-PAGE; gel strips were incubated at 37°C in sodium phosphate buffer, pH 5.0, supplemented with 2 mM DTT. **(C)** Gel strips were also incubated in the absence (control) or presence of cysteine peptidase inhibitors: 1 μM cystatin, 1 μM leupeptin, 1 μM antipain, 1 μM iodoacetamide and 1 μM E-64. Molecular masses, expressed in kDa, are represented on the left. The proteolytic class was determined in cell extracts of parasites grown in BHI medium, but samples from BHI-NTE media were tested in parallel, and similar results were detected.

Our next goal was to evaluate the expression of cysteine peptidases in the different populations through chemical dosages using the fluorogenic peptide substrate Z-Phe-Arg-AMC and gelatin-SDS-PAGE. The data indicated an increase of approximately 1.9- and 2.4-times in the cleavage of the fluorogenic substrate when parasites were cultivated in the presence of NTE at 25% and 50%, respectively, as compared to parasites grown in BHI medium (**Figure 2A**). Corroborating these data, the analysis through SDS-PAGE containing gelatin as substrate revealed the expression of two major bands with molecular masses of approximately 38 and 40 kDa (**Figure 2B**); the densitometric analysis showed that the band intensity was higher in the presence of NTE, indicating a 50% and a 54% increase when parasites were grown in BHI-NTE 25% and 50% versus BHI, respectively. Furthermore, the proteolytic activity bands were inhibited, totally or partially, by different cysteine peptidase inhibitors (cystatin, leupeptin, antipain, iodoacetamide and E-64), allowing to characterize them as cysteine peptidases (**Figure 2C**) as previously described by our group (Santos et al., 2006; Elias et al., 2008; Elias et al., 2009). The proteolytic class was confirmed in cell extracts of parasites grown in BHI medium, but similar results were detected in parasites cultivated in NTE-supplemented media (data not shown).

### Effects of NTE on the Cruzipain-Like Molecules Expressed by *P. serpens*

**Figure 3:**
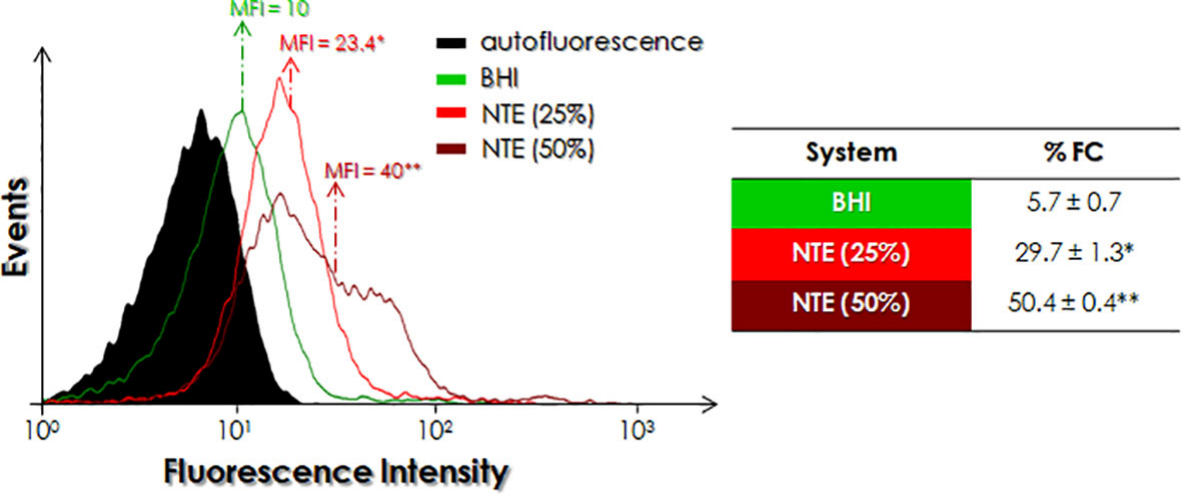
Detection of cruzipain-like molecules in *P. serpens* cultivated in BHI medium supplemented or not with either 25% or 50% natural tomato extract (NTE). Promastigotes were cultured for 48 h and then fixed and processed for flow cytometry analysis using anti-cruzipain antibody. The histogram expresses the mean of fluorescence intensity (MFI) levels. Values represented in the table express the percentage of fluorescent cells (%FC). Each experiment was performed at least three independent times. The symbols indicate the experimental systems considered statistically significant from the BHI medium (*, P < 0.05 and **, P < 0.01; Student’s *t*-test).

**Figure 4:**
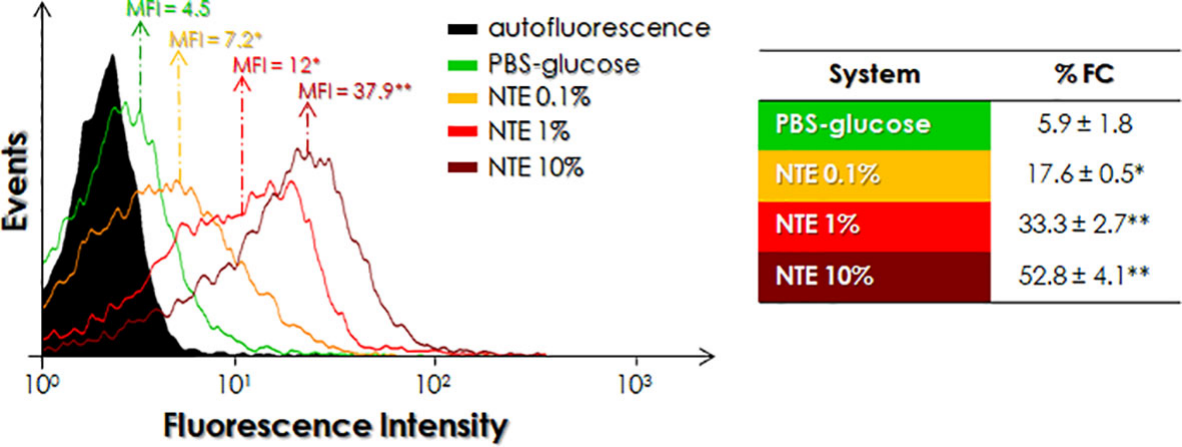
Detection of cruzipain-like molecules in *P. serpens* cultivated in PBS-glucose supplemented or not with 0.1%, 1% or 10% natural tomato extract (NTE). Promastigotes were cultured for 3 h and then fixed and processed for flow cytometry analysis using anti-cruzipain antibody. The histogram expresses the mean of fluorescence intensity (MFI) levels. Values represented in the table express the percentage of fluorescent cells (%FC). Each experiment was performed at least three independent times. The symbols indicate experimental systems considered statistically significant from the PBS-glucose medium (*, P < 0.05 and **, P < 0.01; Student’s *t*-test).

Analysis using flow cytometry showed that parasites cultivated either in BHI-NTE 25 or 50% for 48 h promoted a dose-dependent increase in the expression of cruzipain-like molecules, as can be seen by the increase in both %FC and MFI parameters (**Figure 3**). In this context, the presence of 25% and 50% NTE in the culture medium promoted a 5.2- and 8.8-fold increase in the percentage of positive cruzipain-like cells as well as 2.3- and 4-fold increase in the MFI, respectively, as compared to the growth in BHI medium (**Figure 3**). When parasites were alternatively incubated in PBS-glucose supplemented with different concentrations of NTE for 3 h, a dose-dependent positive modulation in the expression of cruzipain-like molecules was observed (**Figure 4**). When compared to control parasites cultivated in PBS-glucose, *P. serpens* incubated with PBS-glucose supplemented with 0.1, 1 and 10% NTE showed a 3.0-, 5.6- and 8.9-fold increase in the percentage of cells labeled with anti-cruzipain antibody as well as 1.6-, 2.7-, and 8.4-fold increase in MFI, respectively (**Figure 4**).

### Effects of *In Vitro* Infection of Tomatoes on the Cruzipain-Like Molecules Expressed by *P. serpens*

**Figure 5:**
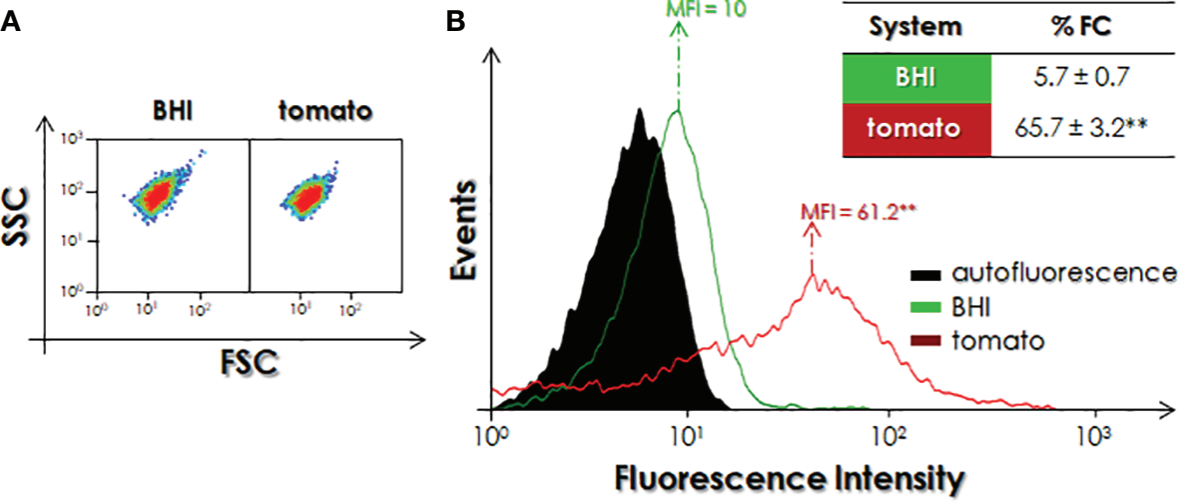
Detection of cruzipain-like molecules in *P. serpens* after infection of tomatoes (*S. lycopersicum*). Promastigotes recovered from infected tomatoes or cultured in BHI medium were washed and analyzed by flow cytometry. **(A)** Morphometric parameters analysis, forward scatter (FSC) and side scatter (SSC); **(B)** Analysis of the expression of cruzipain-like molecules by flow cytometry using anti-cruzipain antibody. The histogram expresses the mean of fluorescence intensity (MFI) levels. Values represented in the table express the percentage of fluorescent cells (%FC). Each experiment was performed at least three independent times. The symbols indicate experimental systems considered statistically significant from the BHI medium (**, P < 0.01; Student’s *t*-test).

Since *P. serpens* promastigotes were able to increase the expression of cruzipain-like molecules when in contact with NTE supplemented in BHI (**Figure 3**) and in PBS-glucose (**Figure 4**) media, we decided to evaluate the modulation of expression of cruzipain-like molecules in parasites recovered from infected tomatoes. In this sense, promastigotes inoculated directly into tomatoes and cultivated for seven days were collected and analyzed using flow cytometry. Initially, we verified whether the cultivation of the parasites in the fruit was able to promote morphological changes. Our data indicated a small reduction in parasite size (FSC = 11.2 ± 0.9) as compared to parasites cultivated in BHI medium (FSC = 16 ± 0.4). Similarly, a small decrease in the granularity parameter was also verified in *P. serpens* cultivated in tomato (SSC = 66 ± 6) as compared to cells cultivated in culture medium (SSC = 76.8 ± 4.7) (**Figure 5A**). Afterward, we evaluated the expression of cruzipain-like molecules on parasites. As verified in the fluorescence intensity histogram, flagellates recovered from the fruit expressed a greater number of molecules similar to cruzipain than control parasites cultivated in BHI culture medium (**Figure 5B**). The data indicate a significant enhancement in both %FC (11.5-fold) and MFI (6.1-fold) parameters from parasites cultivated in tomatoes when compared to control parasites (**Figure 5B**).

### Effects of *In Vitro* Infection of *Oncopeltus fasciatus* on the Cruzipain-Like Molecules Expressed by *P. serpens*

**Figure 6:**
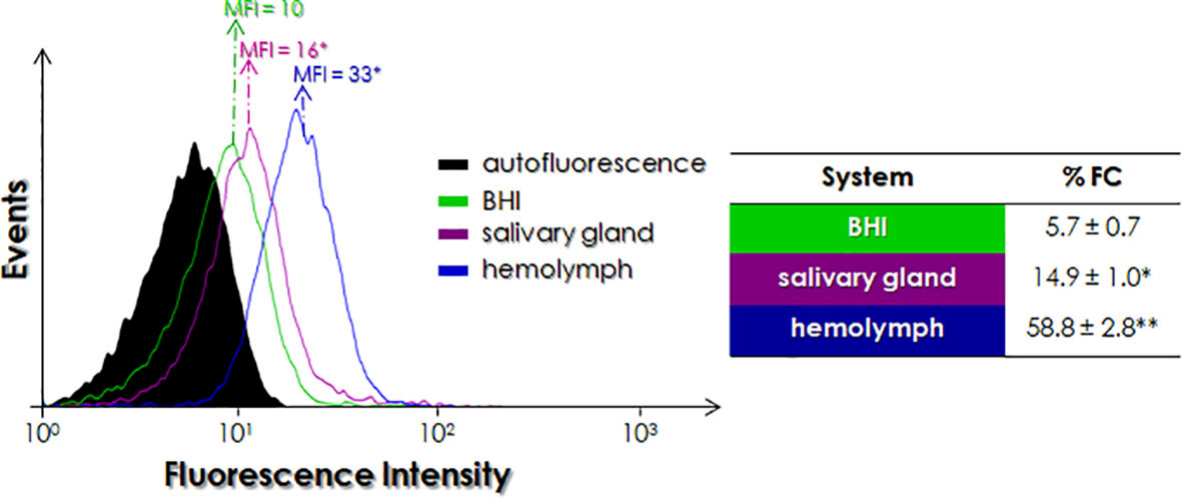
Detection of cruzipain-like molecules in *P. serpens* after infection of *Oncopeltus fasciatus*. Parasites recovered after parasite interaction with explanted salivary glands of *O. fasciatus* and recovered from the hemolymph of infected insects were washed, resuspended in PBS and processed for flow cytometry analysis. The histogram expresses the mean of fluorescence intensity (MFI) levels using anti-cruzipain antibody. Values represented in the table express the percentage of fluorescent cells (%FC). Each experiment was performed at least three independent times. The symbols indicate the experimental systems considered statistically significant from the BHI medium (*, P < 0.05 and **, P < 0.01; Student’s *t*-test).

The expression of cruzipain-like molecules by *P. serpens* was also modulated by *O. fasciatus*. The data represented in the histogram in **Figure 6** indicate a significant increase in MFI both in parasites incubated with dissected salivary glands and in those recovered from infected insects, when compared to *P. serpens* incubated in BHI medium. This augment was more prominent in parasites isolated from hemolymph than in those incubated with salivary glands (**Figure 6**). The percentage of labeled cells was also higher in the parasites isolated from previously infected *O. fasciatus*, when compared to control parasites grown in BHI. There was a 10.3-fold increase in the %FC for cruzipain-like molecules in parasites isolated from hemolymph, while a 2.6-fold increase in the %FC was seen in parasites incubated with the salivary glands.

## Discussion

*Phytomonas serpens* is a heteroxenic parasite that finds extremely different environments during its life cycle: intestine/hemolymph/salivary glands of insects and endocarp of tomatoes; hence, the parasite depends on its adaptation to all these environments in order to proliferate, colonize and survive. In this regard, peptidases are used by trypanosomatids to hydrolyze proteins, allowing the parasite to acquire necessary amino acids to its proliferation as well as to overcome protective barriers of insects and plants, through cleavage of antimicrobial peptides and degradation of cell wall protein components (Mosolov et al., 2001; Mosolov and Valueva, 2004). Production of proteolytic enzymes to degrade host barriers is a common strategy used by microorganisms to colonize tissues and eventually cause diseases; thus, the greater the plasticity of these enzymes, the more effective they will be in host colonization processes.

The first aspect exploited in this work was the parasite ability to survive in BHI culture medium supplemented with natural tomato extract, herein called NTE. We observed that BHI-NTE is favorable for the growth and maintenance of the viability of *P. serpens*, since parasites were able to survive and proliferate without showing any morphological changes. As previously determined by our group, incubation of *P. serpens* in PBS-glucose supplemented with different proteins (BSA, human serum albumin, hemoglobin, mucin, IgG and fetal bovine serum) did not lead to loss of cell viability neither promoted alterations in the measurement of cell size and granularity of the parasites (Elias et al., 2012). In the present study, *P. serpens* was able to grow in a complex medium (BHI) with up to 50% of NTE, although the increase in the concentration of tomato extract resulted in a reduction in the growth rate, probably due to the pH acidification of the culture medium promoted by the addition of NTE and/or due to the dilution of the rich medium (BHI) with the less nutritive NTE.

Jankevicius et al. (1989) had previously infected tomatoes with *P. serpens* and observed that the flagellates were able to multiply actively in the fruits sap; in addition, parasites isolated from the fruit had a smaller body length, body width and flagellum length when compared to parasites cultivated in the culture medium. In this report, similarly, we found that *P. serpens* recovered from infected tomatoes showed a reduction in cell size as compared to parasites grown in BHI medium. In this sense, the great polymorphism of *P. serpens* was previously observed through the presence of two subpopulations of parasites isolated from the digestive tract and salivary glands of the insect vector *P. picta*: one formed by small flagellates and the other one containing large parasites (Jankevicius et al., 1989). In addition, a report showed that 72 h post-infection of *O. fasciatus* with *P. serpens*, 40% of the parasites present in the circulation and almost 100% of the parasites attached to salivary glands of *O. fasciatus* had a long slender cell body (Alves e Silva et al., 2013). Since *P. serpens* has the same evolutionary form throughout its life cycle, the observed size differences according to the host where the parasite is found remains unknown. However, Alves e Silva et al. (2013) proposed that probably the long slender form present in the insect can help the parasite to escape from phagocytosis of hemocytes of invertebrate hosts.

The previous observation by our group that cysteine peptidases from *P. serpens* are able to degrade different proteinaceous substrates reinforces the wide versatility of these enzymes (Elias et al., 2008). In this study, it was possible to observe that expression of cysteine peptidases increases when parasites were cultivated in axenic culture media (BHI or PBS-glucose) containing NTE in a dose-dependent manner. Cysteine peptidases from *P. serpens* have already been biochemically characterized, and identified as homologous molecules to *T. cruzi* cruzipain (Elias et al., 2008; Elias et al., 2009). In *T. cruzi*, this enzyme presents comparable expression levels at all stages of the parasite’s life cycle, although it is found at higher levels in the epimastigote forms, present in the invertebrate host, than in amastigotes or trypomastigotes forms (Branquinha et al., 2015). In epimastigote forms, cruzipain is found in reservosomes, organelles similar to lysosomes, where protein degradation occurs for nutritional purposes. In trypomastigote and amastigote forms, expression of cruzipain is located to the flagellar pocket and cell surface, respectively (Branquinha et al., 2015). In *P. serpens*, cruzipain homologues are located in different cellular compartments, including cytoplasm, membrane, flagellum, and inside flagellar pocket (Elias et al., 2009; Elias et al., 2012).

The increased expression of cysteine peptidases promoted by the presence of NTE in the culture medium was accompanied by an increase in the expression of epitopes reactive to anti-cruzipain antibodies, suggesting the importance of these proteins during establishment of the infection in the fruit host. In the present work, the increased expression of cruzipain-like proteins could also be observed in the direct interaction between *P. serpens*-*S. lycopersicum* (tomato), in parasites incubated with salivary glands of *O. fasciatus* and in parasites recovered from the hemolymph of infected insects. Previously, it was reported that *T. cruzi* isolated from infected *R. prolixus* showed a drastic increase in the expression of surface cruzipain molecules (Uehara et al., 2012). Santos et al. (2006) found that cruzipain-like molecules in *P. serpens* are essential during the invertebrate host life cycle, since treatment of the parasite with either cysteine peptidase inhibitors or anti-cruzipain antibodies promoted a drastic reduction in the interaction process between *P. serpens* and explanted salivary glands of *O. fasciatus*. Interestingly, another report showed that SDS-PAGE analysis using salivary gland extract of *O. fasciatus* as substrate identified a proteolytic activity of cruzipain-like molecules and that these enzymes act by cleaving a 115-kDa polypeptide located on the surface of salivary glands. These data suggest that cruzipain-like molecules are responsible for the cleavage of proteinaceous components present on salivary glands, promoting exposure of key surface receptors for the interaction between *P. serpens* and the insect (Elias et al., 2008). In *T. cruzi*, cruzipain is also crucial for the interaction of the parasite with the invertebrate host, since treatment of *T. cruzi* epimastigotes with anti-cruzipain antibodies or with cysteine peptidase inhibitors (cystatin, antipain, E-64, leupeptin, iodocetamide) decreased the parasite adhesion to *R. prolixus* midgut. Furthermore, mutant epimastigotes overexpressing the endogenous cruzipain inhibitor (chagasin) displayed low rates of adhesion to insect dissected midguts *ex vivo*; in addition, *in vivo* experiments also revealed low levels of colonization of *R. prolixus* midgut and rectum by mutant parasites in comparison to wild-type cells (Uehara et al., 2012).

The acidification of the culture medium containing NTE raised the question as to whether this environment should be considered more hostile for parasite growth. Since an increase in cysteine ​​peptidase activity was also verified in parasites recovered from infected tomatoes, it is possible to correlate this fact to the adaptation to growth in a more acidic medium. Furthermore, it is well reported in the literature that secondary metabolites produced by plants can play a fundamental role in the resistance of plants to parasitic infections (Friedman, 2002; Evangelista et al., 2018). For instance, the glycoalkaloid α-tomatine has a potent antimicrobial action and it is found mainly in green tomatoes, with decreased concentration during fruit maturation (Medina et al., 2015). α-tomatine and its aglycone structure, tomatidine, display *in vitro* toxicity against *P. serpens*, but the lower levels found in mature tomatoes are not able to act as an effective barrier against *P. serpens* infection (Medina et al., 2015). However, prolonged exposure time to tomatidine may decrease the parasite’s growth (Evangelista et al., 2018). Additionally, peptidase inhibitors are also produced by tomatoes in order to protect the plant against pathogens and predators (Friedman, 2002). In this sense, the increased expression of cysteine ​​peptidases may also be acting as a compensatory mechanism due to the production of peptidase inhibitors, ensuring parasite survival.

The knowledge about the metabolism of *P. serpens* is essential to understand its pathological potential for tomato fruits, and the proteomic analysis of the parasite allowed the identification of enzymes of metabolic pathways located in glycosome, mitochondrion and cytosol (Dos Santos Júnior et al., 2018). However, no enzymatic activity involved in the hydrolysis of polysaccharides into monosaccharides, such as amylases, amylomaltases, invertases or carboxymethylcellulases, were identified in *P. serpens*. On the other hand, peptidases present in these parasites seem to be aid the establishment of the infection in the fruit and in the invertebrate host. In this report, we verified that cysteine peptidases can help in different processes, either in the degradation of protein substrates that will provide necessary amino acids for their proliferation and nutrition, or during the interaction with the fruit and insect contributing to the survival of the parasite in these environments. Altogether, our data indicate that cruzipain-like molecules are expressed throughout the life cycle of *P. serpens*, playing essential roles in the parasite’s interaction processes with the invertebrate as well as with plant hosts, which must be further explored.

## Data Availability Statement

The original contributions presented in the study are included in the article/supplementary material. Further inquiries can be directed to the corresponding authors.

## Author Contributions

All authors conceived and designed the experiments. SO, CE, and FD performed the experiments. All authors analyzed the data. AL, CD’A-L, AS, and MB contributed reagents/materials/analysis tools. All authors wrote and revised the paper. All authors contributed to the research and approved the final version of the manuscript. All authors agree to be accountable for all aspects of the work. All authors have read and agreed to the published version of the manuscript.

## Funding

This research was funded by grants from Conselho Nacional de Desenvolvimento Científico e Tecnológico (CNPq), Coordenação de Aperfeiçoamento de Pessoal de Nível Superior (CAPES – Finance Code 001), Fundação Carlos Chagas Filho de Amparo à Pesquisa do Estado do Rio de Janeiro (FAPERJ) and Fundação Oswaldo Cruz (FIOCRUZ).

## Conflict of Interest

The authors declare that the research was conducted in the absence of any commercial or financial relationships that could be construed as a potential conflict of interest.

## Publisher’s Note

All claims expressed in this article are solely those of the authors and do not necessarily represent those of their affiliated organizations, or those of the publisher, the editors and the reviewers. Any product that may be evaluated in this article, or claim that may be made by its manufacturer, is not guaranteed or endorsed by the publisher.
